# Supplementary material for: TAPES: A tool for assessment and prioritisation in exome studies
Source: PLoS Comput Biol. 2019 Oct 15;15(10):e1007453. doi: 10.1371/journal.pcbi.1007453 (PMC6814239; doi:10.1371/journal.pcbi.1007453)
Supplement: Attachment — Submitted filename: PlosCompBiol Response_BTP_RJS.docx [file pcbi.1007453.s006.docx]

**Alexandre Xavier**

PhD Candidate

The University Of Newcastle, School of Biomedical Science and Pharmacy, Australia

**Re: Manuscript PCOMPBIOL-D-19-01091**

Thank you for your email on the 2^nd^ August 2019 regarding the resubmission of PCOMPBIOL-D-19-01091 entitled “TAPES: a tool for assessment and prioritisation in exome studies”. The manuscript has been revised taking into account the minor/major revisions indicated by the reviewers and should now be acceptable for publication in PLOS Computational Biology.

The manuscript has been appropriately altered according to reviewers’ comments and I will, in point form, describe what has been edited.

**Reviewer #1: SUMMARY:

'TAPES: a tool for assessment and prioritisation in exome studies' implements a novel and more precise method for assessing variant pathogenicity by introducing a novel modelling for integration of ACMG criteria. They leverage this model along with publicly available variant population frequencies to provide more accurate predictions of variant pathogenicity. Additionally, this software provides a comprehensive list of both reporting and analysis options.


MAJOR CODE PROBLEMS:
- Code doesn't seem to have any tests or automated way to run them: https://github.com/a-xavier/tapes. Please add tests (preferably using a testing framework such as PyTest) that minimally take advantage of your toy datasets that covers most of your functionality. Integration with a free and automated continuous integration environment like Travis would also be highly recommended. Once tests are in place, potentially using branches to provide a more stable development path may aid development**

Author’s Answer: We linked the TAPES github repository to Travis for continuous integration (https://travis-ci.org/a-xavier/tapes). As a test framework, we used a simple test function that covers the main report creation and all of TAPES available sort options on a small dataset (rather than Pytest that would require a lot of code refactoring). Once Travis testings were successful, we branched the Master branch to a Testing branch for future updates and to keep Master stable.

Travis integration also showed that TAPES can be run in a virtual environment out of the box (On Linux, Windows and MacOs). The ‘Availability and future direction’ section has been modified accordingly (line 217). **- Toy example provided doesn't work natively or within a virtualenv:
- python3 tapes.py sort -i ./Example_Output/input.csv -o ./Toy_dataset/ --tab --by_gene --by_sample --enrichr --disease "autosomal dominant" --kegg "Pathways in cancer":
No acmg_db path given and no db_config.json found
Default is: /home/ubuntu/repositories/tapes/acmg_db

***TAPES: SORT***

2019-07-15 10:37:05.....Output type: FOLDER
Traceback (most recent call last):
File "tapes.py", line 309, in
main()
File "tapes.py", line 164, in main
output_prefix = args.output.split('\\')[-2]
IndexError: list index out of range**

Author’s Answer: Unfortunately, this bug was caused by code expecting backslashes ‘\’ as folder separator in windows and slashes ‘/’ in Linux. It is now fixed by detecting the type of separator and acting accordingly regardless of the user’s operating system.

In addition further instructions for installation in a virtual environment was added as well as differentiated dependency requirements for windows users as the module ‘pysam’ (used for bcf input annotation required only for ANNOVAR integration) is unavailable for windows.  **MINOR CODE FEEDBACK
- I would put code that is not top-level in a `src` or `source` directory.**

Author’s Answer: all the code outside of the main tape.py script has now been moved to a ‘src’ folder.

**- While the Manual is fine as a PDF, long-term maintenance might be easier if it is in markdown. It can be further extrapolated using something like Read the Docs or other services.**

Author’s Answer: The markdown version of the manual was made available on github. In addition, all information contained in the manual is also present on the Github Wiki (<https://github.com/a-xavier/tapes/wiki>) see ‘Availability and future direction’ line 215.

**MINOR EDITS:
- line 25: should be: "share the same phenotype" , missing "the"**

Author’s Answer: Sentence modified to ‘sharing a common phenotype (now on line 26) ’

**- line 27: I think it reads better to say "Benchmarks showed that TAPES outperforms available tools"**

Author’s Answer: The sentence was modified as suggested (now on line 28).

**- line 34: "Available software can predict" drop "'s"**

Author’s Answer: The extra‘s’ was removed to read: *“Available softwar****e*** *can predict”* (now on line 35).
 **- line 90: "individuals affected" and "number of individuals", individuals I believe should be plural in both cases**

Author’s Answer: The sentence was corrected to *“the number of individual****s*** *affected by the variant in the control population and* $N_{c}$ *is the number of individual****s****”* (now on line 93)

**- line 96: "very rare variants" I believe variants should be plural**

Author’s Answer: The sentence was modified as suggested (now on line 99).

**- line 134: "cohort are in the class are probably" , missing "are"**

Author’s Answer: The sentence was removed based on further comments.

**- line 137-139: This is not a complete sentence.**

Author’s Answer: The sentence was reconstructed to read: “*In some cases, poorly mapped reads (for example due to pseudo-autosomal regions in the* X or Y chromosome*), might impact the result with an excessive number of samples affected by a variant*” (now on line 143-145)

**- Figure 1: Charger should be "CharGer" in your legend**

Author’s Answer: Figure 1 legend was modified to read “CharGer” instead of “Charger” **MINOR QUESTIONS:
- In this model there are no controls, which is novel. I'm mildly curious if it can be shown that providing controls offers little or no statistical benefit over the publicly available variant frequencies.**

Author’s Answer: Providing controls would make little difference. gNomad provides 125,748 exomes and 15,708 genomes sequenced across a spectrum of ethnicities, making it one on the most representative control populations.

Providing controls would yield comparable Minor Allele Frequencies (MAFs) to gNomad or ExaC MAFs and probably be overall less accurate. TAPES PS4 criteria assignment was mostly designed to catch the enrichment of rare variants, which mean that, in most cases, the control population will either have 0 or only 1 sample affected unless the scale of the control population is significantly increased.

If we take the example of a rare variant of frequency: 0.0005 (5e^-4^) from gNomad.

Using 1000 control samples would either yield 0 or 1 affected individual. There is a 39% chance that at least 1 individual out of the 1000 will have the variant (1 - (1 - 0.0005)^1000^ = 0.393) and 61% chance that no individual will carry the variant ((1 - 0.0005)^1000^ = 0.6064). (see graph below)

If this particular variant is enriched in the case cohort being studied (for example 2 individuals out of 50 with the variant):

OR = 41.6250, P = 0.0025 with 1 sample affected in the control population.

OR = 103.1443, P = 0.0029 with 0 samples affected in the control population.

Using TAPES extrapolation of MAF from gNomad: OR = 41.6250 and P < 0.0001.

In all cases, the enrichment for the variant was detected accordingly.

**- A minor discussion of why the CharGer Scores were so similar to the TAPES probability model might be useful in context of Figure 1.**

Author’s Answer: CharGer is a very precise and specific tool. It will only assign pathogenicity for variants with a high custom score.

The dataset used for benchmarking TAPES was the same used for the CharGer’s own publication. To work best, CharGer needs user provided files. Those files suggest that CharGer needs the user to have prior assumptions about the cohort studied.

In their own publication they supplied several additional files for their analysis.

“For the purpose of this analysis of cancer predisposition, we supplied additional input files to CharGer as follows: 152 cancer predisposition genes (Huang et al., 2018), 1819 cancer predisposition variants as curated by the TCGA PanCanAtlas Germline Analysis Working Group (Huang et al., 2018), and 17, 555 somatic mutation clusters computed from the TCGA MC3 MAF (Ellrott et al., 2018) using HotSpot3D (ver. 1.1.4, with the default settings except 10 Å cutoff and 20 Å maximum radius). We also used a tab-delimited ClinVar flat file (accessed 30 October 2017 from github.com/macarthur-lab/clinvar)” *from Scott AD, Huang KL, Weerasinghe A, Mashl RJ, Gao Q, Martins Rodrigues F, et al. CharGer: Clinical Characterization of Germline Variants. Bioinformatics. 2018.*

On the other hand both InterVar and TAPES do not make any assumption on the type of cohort (such as which genes should be scored higher than any other for the same criteria) and do not require additional user-provided inputs (other than the annotated VCF file). This explains why, when using the ACMG criteria, the Area Under the Curve and Average Precision are similar.

During our own tests, using CharGer without any of those additional files and using only a single VEP annotated VCF as input only resulted in every single variants being classified as “Uncertain Significance ‘’.A sentence has been added to reflect these facts (line 176).

**Reviewer #2: The article "TAPES: a tool for assessment and prioritisation in exome
studies" describes a new software tool to identify pathogenic and
benign variants. The aim described is very promising. However, I think
the clarity of both the paper and the documentation could be improved.
I will first comment on my experience with the software and then on
the paper. (This review is writen in MarkDown format, so it can be
converted to html or other format to see code section.)

\## Comments on the software package.
I cloned the repository from GitHub, and could install it. Then I ran
into a few problems.
1. I found a bug in `t_func.py` on line 3197 that made the program
crash
```
with gzip.open(os.path.join(acmg_db_path, 'repeat_dict.{}.gz'.format(assembly)), "r") as dj:
```
Correcting the line to the following solved the issue.
```
with gzip.open(os.path.join(acmg_db_path, 'repeat_dict.{}.gz'.format(assembly)), "rt") as dj:
```**

Author’s Answer: The bug was corrected and included in the further commits on github.

**2. It was not clear at installation that I should install annovar to
be able to use tapes. I have found this information later in the
manual.**

Author’s Answer: It is clarified on the github readme file that ANNOVAR wrapping is totally optional.

**3. After installing annovar I needed to run
`python3 tapes.py db -s -A annovar` and
`python3 tapes.py db -b annovar` before I could annotate vcf
files. These commands were only mentioned at the end of the manual.**

Author’s Answer: The workflow example on the wiki (<https://github.com/a-xavier/tapes/wiki/Workflow>) has now been added earlier in the manual to better explain the TAPES workflow and commands.

**4. I did not manage to find a way to start with a vcf file, annotate
it and finally obtain ACMG classification. I think a tutorial and an
example dataset (starting from vcf files) would be valuable additions.**

Author’s Answer: A new toy dataset was added and the ‘Example_Output’ folder was removed from the repository.

The new toy dataset is still based on Zhang et al 2015. It is composed of an unannotated vcf file containing all variants evaluated by Zhang et al 2015. Single and multi-sample annotated (with ANNOVAR and VEP) vcf files are also present to test all TAPES functions.

This makes it easier for new user to both test the full workflow or just the sorting option.

The new toy dataset is also accompanied by a readme file that explains the main workflow.

**5. I would suggest to add a workflow diagram both to the manual and to
the paper to make it clear what kind of steps are needed and what
are the potential input and output files.**

Author’s Answer: The manual and the paper have been updated to explain better what TAPES can do and how it is used.

**6. I could not identify what was the input file used for the analysis
shown in the paper, so I could not check whether it is
reproducible.**

Author’s Answer: The original input file was not provided with the paper. We only provided a file comparing the results from different software (Supplementary_Table_2_comp_soft.xlsx).

The original file used for the analysis was the supplementary figure from the CharGer manuscript (supplementary table 2 from the CharGer article) (itself derived from Zhang et al 2015). The chromosome, position, reference allele and alternative allele were extracted to create a vcf file.

This unannotated vcf file is the example toy dataset (https://github.com/a-xavier/tapes/blob/master/toy_dataset/toy.vcf).

This vcf file was then analysed by both InterVar and TAPES for comparison purpose and we compared it to the CharGer publication results (see supplementary table 2 from TAPES for the comparison between the 3 software)

We will upload all those files (CharGer, InterVar and TAPES analysis) for review (respectively called TAPES_toy_analysis, CharGer_toy_analysis (which is extracted from the CharGer publication) and InterVar_toy_analysis).

**7. The program does not always produce the expected file name or it
does create the expected file, but does not log it
correctly. I think the code needs to be checked more thoroughly.**

Author’s Answer: We were unable to reproduce this behaviour.

However, TAPES does append suffixes to filenames during analysis (to differentiate the type of report generated). The github wiki and manual were updated to better explain this fact.

**8. Please create a release for the publication version of the package
so people can know which version/status of the software was used
for the publication (This can be done at** [**https://github.com/a-xavier/tapes/releases**](https://github.com/a-xavier/tapes/releases)**).**

Author’s Answer: A first release version was created (0.1) to mark the beginning of the release cycle. A sentence was added to the ‘AVAILABILITY AND FUTURE DIRECTIONS’ section (line 216).

**9. A docker image is always a nice addition, to make sure that
everything is specified as it should be, and there are no problems
due to difference in the software environment. It is also a good
way to test, how a software can be installed in a new environment.**

Author’s Answer: The creation of a Docker image was added to the list of features to add. Travis CI showed that TAPES can indeed be installed in all major operating systems.  **\## Comments on the paper
I have found several typos and grammatically mistakes. I think the
text should be checked more thoroughly for mistakes.

1. Abstract line 17: What does "downstream" variants refer to?**

Author’s Answer: The sentence was corrected to ‘better predict variant effects in downstream analysis’.

**2. On line 20 multi-sample variant calling formats are mentioned in
the abstract, but this is never mentioned further in the article. I
would either remove it from the abstract or add an explanation to
a later section.**

Author’s Answer: One of TAPES main feature is its ability to handle multi-sample vcf to calculate enrichment. This requires multi-sample vcf. A sentence was added to line 84 to better reflect this fact.

**3. Lines 25-26. The Authors mention that cohort samples can be analyzed
even without a control sample set. My question is whether it is
possible to make use of a control set or is it only possible to use
the standard option where the databases are checked?**

Author’s Answer: For now it is only possible to use TAPES without controls. The ability to use real controls was added to the list of future features to add.

**4. Lines 26-27: "Finally, it can provide powerful filtering and
reporting options to help researchers make sense of cohort
studies." I would say "it provides powerful filtering and
reporting options". I find "make sense" to be too informal for a
scientific paper.**

Author’s Answer: The last part of the sentence was deleted.

**5. The Author summary contains several typos also some have
grammatical mistakes as well.**

Author’s Answer: The summary was modified for better clarity. Additionally, all spelling and grammatical errors were corrected.

**6. Lines 34-35: "but does not take into account the fact that the
variants belongs in a cohort." I don't know what this sentence
refers to exactly. Also, I have the same comment for line 52: "any
cohort characteristic". I think there should be a clear discussion
on what these are and how they are used or not used by the
different software tools.**

Author’s Answer: The sentence in line 35 was corrected to “does not take into account the abundance of a variants in a cohort” and line 52 to “they do not have the ability to take into account the frequency of variants in a cohort”.

**7. ANNOVAR interface and annotated variant file: lines 66-69. This
section contains grammatical mistakes and is not clearly
structured. I think it would be good to have workflow chart to make
clear how different inputs can be used. Starting from VCF either
VCF --(3rd party tools)--> annoteted VCF or
VCF --(TAPES as a wrapper for ANNOVAR)--> annoteted VCF. And how to proceed
with the annotated VCF. I could not use the sort function on a VCF,
only on CSV.**

Author’s Answer: The manual has been updated with a simple ‘choose your own starting point’ and a workflow corresponding. In addition, the toy dataset now includes an unannotated vcf as well as a VEP and ANNOVAR annotated vcf rather than only a csv file (to easily test the ANNOVAR wrapping functions).

**8. Line 69: "without having to specify the databases and annotations
to use." It is true that when running one does not have to specify them,
but at set up the user has to specify which databases are to be
used, according to my experience. Also this comment gives the
impression that the user has no control over which databases are
being used.**

Author’s Answer: TAPES can be used as a wrapper for ANNOVAR. Two different modes are available: a simple and an ‘advance’ one.

If the user just need to annotated their VCF file to use with TAPES,the simple mode allows one to just use the ‘annotate’ function with a vcf input and the ‘--acmg’ tag.

Otherwise, users have full control of the databases used for annotation using the two files created at first use: db_config.json and db_vcf.json representing respectively the databases already downloaded by the user and the databases that the user wants to use for annotation. All ANNOVAR databases are available.

The manual and wiki have been updated to better reflect these functionalities.

**9. Lines 76-80. I think it would be nice to have a description on how
each criterion was implemented as supplementary at least. Then the
Authors could say that most criteria were straight forward to
implement (see suppl.), but the others we solved in the following
way, and then explain them.**

Author’s Answer: These details were previously implemented in the Manual as an appendix. It will now be uploaded during the submission as a supplementary file (Supplementary_table_1_Assignment.xlsx) and referred to in the manuscript (line 81).

**10. Line 95. Assumptions "are" made.**

Author’s Answer: The sentence was corrected.

**11. Line 125. I would not use "most confidence", but "highest level of
confidence".**

Author’s Answer: Agree and corrected

**12. Lines 128-129. I would suggest to reformulate the first sentence
to make it clearer.**

Author’s Answer: The sentence was replaced to better explain what the gene burden score represents.

**13. Line 134. What does "in the class" mean?**

Author’s Answer: The sentence was modified for a previous comment. (see Reviewer 1, minor comments )

**14. Line 169. "sheer number of" I find this a bit too informal.**

Author’s Answer: “Sheer number” was replaced by ‘absolute number’.

**15. Figure 1. and surrounding text is not well formulated. It is
difficult to interpret the difference between "TAPES proba" and
"TAPES ACMG". I have only realized what the difference was once I
opened the supplementary table and saw the last two columns. I
think this could be improved.**

Author’s Answer: This is unfortunate, Figure 1 legend has been modified to better explain the terms TAPES proba and TAPES ACMG.

**16. Lines 166-167. How did the Authors arrive at the threshold values (0.35 and 0.8) for
probability scores? Was it to maximize the score on the
example/training dataset? I would suggest to use more than one dataset
for benchmarking and testing. It should be avoided to optimize a
method on the benchmarking set.**

Author’s Answer: The threshold of 0.35 for benignity and 0.8 for pathogenicity was a simple indication. We did not ‘use it’ during benchmarks but rather the ROC curves showed us that it was the most performant threshold (since ROC curves tests specificity and sensitivity for all thresholds).

**17. Figure 1. According to the benchmarks TAPES falls either between the two other
software or performs worse than the other two software if we use the
ACMG results according to the ROC and precision recall analysis. While
this is not discussed in the text. Also would the Authors suggest to
use the Probability instead of ACMG then?**

Author’s Answer: Please refer to reviewer 1 minor question 2. Yes using the probability prediction is the preferred way of analysing TAPES reports.

Here is a copy of the answer.

CharGer is a very precise and specific tool. It will only assign pathogenicity for variants with a high custom score.

The dataset used for benchmarking TAPES was the same used for the CharGer’s own publication. To work best, Charger needs user provided files. Those files suggest that CharGer needs the user to have prior assumptions about the cohort studied.

In their own publication they supplied a number of additional files for their analysis.

“For the purpose of this analysis of cancer predisposition, we supplied additional input files to CharGer as follows: 152 cancer predisposition genes (Huang et al., 2018), 1819 cancer predisposition variants as curated by the TCGA PanCanAtlas Germline Analysis Working Group (Huang et al., 2018), and 17, 555 somatic mutation clusters computed from the TCGA MC3 MAF (Ellrott et al., 2018) using HotSpot3D (ver. 1.1.4, with the default settings except 10 Å cutoff and 20 Å maximum radius). We also used a tab-delimited ClinVar flat file (accessed 30 October 2017 from github.com/macarthur-lab/clinvar)” *from Scott AD, Huang KL, Weerasinghe A, Mashl RJ, Gao Q, Martins Rodrigues F, et al. CharGer: Clinical Characterization of Germline Variants. Bioinformatics. 2018.*

On the other hands both InterVar and TAPES do not make any assumption on the type of cohort (such as which genes should be scored higher than other for the same criteria) and do not require additional user-provided inputs (other than the annotated VCF file). This explains why the use if ACMG criteria for Area Under the Curve and Average Precision are similar.

During our own tests, using CharGer without those additional files and using only a single VEP annotated VCF as input only resulted in every single variants being classified as “Uncertain Significance ‘’.A sentence has been added to reflect these facts line 173.

**18. Figure 3. I prefer graphs with two axes. Having two y-axes makes
it difficult to interpret. The two graphs could be shown below each
other (A and B) with the same x-axis, but separate y-axes for the two plots.**

Author’s Answer: Figure 3 was modified accordingly for better clarity.

**19. Lines 188-194. I think this section (“Variant enrichment / PS4 Benchmark”) could be improved by adding example output, adding context on how does it compare to a
workflow without TAPES to fully show the benefits of the method.
I suggest to separate real data and mock up (made up) data
examples.**

Author’s Answer: To our knowledge, no software predicting variant pathogenicity can handle multi-sample vcf and thus cannot assign PS4 and calculate enrichment.

PS4 is a strong evidence for pathogenicity, it can highlight variant that would not otherwise be considered without taking into account the frequency of the variant If we consider the example of:

| Chr | Start | End | Ref | Alt | Gene |
| --- | --- | --- | --- | --- | --- |
| 2 | 48027746 | 48027746 | T | C | MSH6 |

This variant is considered as ‘Uncertain significance by InterVar, CharGer and TAPES with a TAPES probability of pathogenicity of 0.6752 (with PM1, PM2, PP3 and BP1 assigned). It has a maximum frequency of 0.00009241 (9.24e^-5^), with 2 heterozygous individuals in 10821 individuals (gNomad exome).

PS4 could be assigned to this variant if it was found in only 2 case individuals out of 500 (OR = 21.7, p = 0.0021). It would change this variant classification to ‘Pathogenic’.

This shows how important it is to take into account the frequency of variants in a cohort. A workflow without using TAPES would have missed such variant.

**20. TAPES is able to assign variants to ACMG categories and then can
do further sorting and reporting. Other software tools can also
use ACMG categories as mentioned in the introduction. Can TAPES
use the output of those software and then do sorting and reporting?**

Author’s Answer: Most of the downstream analysis from TAPES are specific to TAPES main report output. It requires keeping individual genotyping which most software do not offer.

However InterVar output could be formatted to be compatible with TAPES reporting options as they use similar inputs. CharGer reports, on the other hand, rely on a custom score and would be harder to adapt.

The ability to use InterVar outputs with TAPES was added to the list of future features to add.

**21. On which platforms was TAPES tested?**

Author’s Answer: TAPES was thoroughly tested on Windows 7 and different flavours of Linux (mainly Fedora 30, Manjaro and Ubuntu 18.10).

MacOs was not tested due to the lack of proper access to it but all tests on Travis CI were successful.

This information was added to both the github wiki and the manual.

**22. Please add a release for TAPES that is referred to in the article.
Also add version numbers for the software used (or commit tags from GitHub).**

Author’s Answer: See Reviewer #2 question 8 in the section “Comments on the software package**”.** A first release version was created (0.1) on github and will be referred to in the manuscript (line 216).

**\## Summary
Overall, I find the tool promising. However, I do think both the
software package and the article require significant revision.
I do believe that TAPES can become a valuable tool.**

I appreciate all the helpful comments from the reviewers and trust the manuscript is ready for publication in PLOS Computational Biology.

Kind regards,

Alexandre Xavier

PhD Candidate (Medical Genetics)

University Of Newcastle

School of Biomedical Science and Pharmacy
