# Supplementary material for: TAPES: A tool for assessment and prioritisation in exome studies
Source: PLoS Comput Biol. 2019 Oct 15;15(10):e1007453. doi: 10.1371/journal.pcbi.1007453 (PMC6814239; doi:10.1371/journal.pcbi.1007453)
Supplement: Attachment — Submitted filename: PlosCompBiol Response_2.docx [file pcbi.1007453.s007.docx]

**Alexandre Xavier**

PhD Candidate

The University Of Newcastle, School of Biomedical Science and Pharmacy, Australia

**Re: Manuscript PCOMPBIOL-D-19-01091R1**

Thank you for your email regarding the resubmission of PCOMPBIOL-D-19-01091 entitled “TAPES: a tool for assessment and prioritisation in exome studies”. The manuscript has been revised taking into account the minor/major revisions indicated by the reviewers and should now be acceptable for publication in PLOS Computational Biology.

The manuscript has been appropriately altered according to reviewers’ comments and I will, in point form, describe what has been edited.

**Reviewer #1: Thanks you for addressing my concerns.

Reviewer #2: In my opinion the manuscript is clearer now thanks to the corrections. I still think that adding a flowchart on input, output and processes could be very helpful to easily understand the pipeline and the possibilities. Below I have included a suggested workflow chart.

Most importantly, please indicate clearly the input file and the reference set for the benchmark, and how the thresholds were calculated. Otherwise, readers cannot evaluate the validity, it would be only faith or distrust.**

Author’s answer: Here is how we benchmarked TAPES:

- **Choosing the dataset**

We used the dataset from Zhang et al. 2015 (Zhang J, Walsh MF, Wu G, Edmonson MN, Gruber TA, Easton J, et al. Germline Mutations in Predisposition Genes in Pediatric Cancer. N Engl J Med. 2015;373(24):2336-46.) because hand curated pathogenicity prediction from panel expert are quite rare.

The most useful database of curated variant would be Clinvar but as we already use it as a criteria to predict pathogenicity, it would integrate bias in the benchmark.

- **CharGer Benchmark**

The second advantage is that CharGer used the same dataset to benchmark their tool which makes comparison easier.

The supplementary table 2 of CharGer’s publication was the starting point of our benchmark. It includes:

- the decision from the expert panel (from Zhang et al 2015)

- the pathogenicity prediction from CharGer (with their custom score)

This unmodified file was previously uploaded as *Charger_toy_analysis.xlsx*. We reuploaded it with the name **CharGer_and_Panel_Benchmark.xlsx** for clarity. A line has been added line 158 to clarify that the same dataset has been used for TAPES benchmark.

- **Creating a synthetic VCF**

The information from **CharGer_and_Panel_Benchmark.xlsx** (chromosome, position, reference and alternative allele) were extracted to create a synthetic vcf file. It contains all the information from the variants present in the original file in the vcf format.

It is the file called toy.vcf in the github repository (as indicated in the previous response).

We reuploaded it with the name **Synthetic_VCF_for_Benchmark.vcf.**

- **InterVar Benchmarking**

We used the **Synthetic_VCF_for_Benchmark.vcf** with InterVar (following the standard instructions).

InterVar’s output containing InterVar’s pathogenicity prediction and ACMG classification was reupload with the name **InterVar_Benchmark.txt** (it was previously uploaded as InterVar_toy_analysis.txt).

- **TAPES Benchmarking**

We used **Synthetic_VCF_for_Benchmark.vcf** with TAPES (both annotation and pathogenicity prediction).

TAPES’ output containing TAPES’ pathogenicity prediction, ACMG classification and probability of pathogenicity was reuploaded with the name **TAPES_Benchmark.xlsx** (it was previously uploaded as TAPES_toy_analysis.xlsx).

- **Aggregating all benchmarks**

For each variant, we created a table containing:

- the variant’s informations

- the expert panel decision on pathogenicity

- CharGer predictions

- InterVar predictions

- TAPES prediction and probability

This tables was uploaded as Supplementary_Table_2_comp_soft.xlsx. It was reuploaded as **Supplementary_Table_2_Benchmark_software_comparison.xlsx** for clarity.

We then plotted ROC curves and Recall Precision curves using this table.

Based on further comments, we now used a different validation dataset (see answers below) and will recommend two sets of thresholds (lenient and strict) for pathogenicity and benignity.

**In addition, I recommend to further improve the git repo, because potential users will give up easily if it is not clear or there are too many mistakes. Adding a tutorial with example input (e.g. the benchmark would be an excellent example), commands to run and the interpretation would help users test that everything is installed correctly and help them understand how the program works and what the steps are.**

Author’s answer: The toy dataset present in the github repository was already the one we used for the benchmark (we added this statement on the readme in the toy-dataset folder) and example commands are present in the toy_dataset folder.

There are both annotated and non-annotated vcf present in the toy dataset to test all functionalities of TAPES.

To reflect this fact more clearly, we changed completely the github readme/front page to include a tutorial section and a clearer explanations on TAPES purpose. **Example flowchart, based on the manual, in mermaid (it can be drawn using the online editor: https://mermaidjs.github.io/mermaid-live-editor/):
```
graph TD
VCF -->an{annotate}
VCF -->an2
subgraph ANNOVAR
an2{annotate: table_annovar.pl}
end
an2 -->VCF2[VCF: annotated variants]
an2 -->TSV[TSV: annotated variants]
VCF2-->an
TSV -->|?|sort
subgraph TAPES
subgraph wrapped ANNOVAR
an
end
an --> Annot[CSV: annotated variants]
Annot -->sort{sort}
sort -->Sorted[CSV: sorted varainats]
Sorted -->X{analyse}
end
X -->|by_sample| S[By sample report]
X -->|by_gene| G[By gene reoprt]
X -->|enrich| E[EnrichR report]
X -->|list| L[Kegg, List and Disease reoprt]
```**

Author’s answer: Thank you for the suggestion, a slightly modified version of this flowchart has been added to the front page (readme) of the github repository to better explain TAPES workflow.

**\# Questions based on the response from the Authors

In one of the answers the Authors mention that for the benchmarking they used the dataset from the CharGer publication. Please include this also in the manuscript, and also add to the repository.**

Author’s answer: A sentence has been added on line 158 to better reflect the fact that we used the same dataset as CharGer for benchmarking TAPES. It was also added in the toy dataset folder readme.

**The same answer ends with the comment that a sentence has been added to line 176. I think that line numbering has changed, so I could not find the referenced sentence.**

Author’s answer: The sentence was moved to line 169-173. This sentence was to address the fact that CharGer scored better than TAPES ACMG prediction. **The Authors claim that ANNOVAR wrapping is totally optional, although I could not run any of the commands without setting up the database by first installing ANNOVAR.**

Author’s answer: The main sorting function does not require ANNOVAR installed to work (all builds are passed on Travis CI without the need of ANNOVAR). However, we made clearer that the ANNOVAR wrapping and annotation step requires ANNOVAR to work (line 68). **I still don't understand how the threshold values 0.35 and 0.8 were chosen for the probability method which is the recommended method. My assumption is that based on the benchmark set the Authors identified which cutoffs would yield the maximum number of correctly identified variants. If this is true then an independent data set is needed to test how valid the calls are, because the benchmark and training set should be independent from each other. If this assumption is false, please include the method used for deciding the threshold values.**

Author’s answer: We indeed extrapolated the thresholds (0.8 for pathogenicity and 0.35 for benignity) using the initial benchmark as we did not feel that it would affect the prediction power of TAPES.

(See answer below to the question: **Line 167-168. How do the ROC curves suggest the threshold values?**)

However, having a validation dataset is a valuable addition.

We now include a validation dataset to better test the prediction using the proposed thresholds. This new dataset comes from ClinGen (https://www.clinicalgenome.org/) which is an entity offering hand curation of variant to assess their pathogenicity. Panels of expert assigns pathogenicity related to a specified disease.

To do so, we downloaded the evidence repository, a curated list of variants from ClinGen (<https://erepo.clinicalgenome.org/evrepo/>). We extracted 530 variants with a clear ALT and REF allele to create a synthetic vcf file.

This VCF file was processed using CharGer, InterVar and TAPES. We then compared the pathogenicity prediction from experts to the predictions from CharGer, InterVar and TAPES.

Using CharGer on default settings, even after using the additional files present on the github repository (*152 cancer predisposition genes (Huang* et al.*, 2018), 1819 cancer predisposition variants as curated by the TCGA PanCanAtlas Germline Analysis Working Group (Huang* et al.*, 2018), and 17, 555 somatic mutation clusters computed from the TCGA MC3 MAF (Ellrott* et al.*, 2018) using HotSpot3D*), the results were quite poor, with only a handful of variants classified as pathogenic. Even after providing 41 additional disease predisposition genes along with their mode of inheritance and gene lists for PP2 and BP1 criteria (gene with many pathogenic missense variants or low pathogenic missense variants), yielding very few variants benign or pathogenic. We believe that CharGer might be geared toward cancer rather than a general disease tool.

Using the results from this validation dataset, we now propose two different thresholds for pathogenicity and benignity, 0.80 and 0.35 as lenient thresholds and 0.85 and 0.20 as strict thresholds. See line 184.

However, the general trend that the probability of pathogenicity using TAPES is the best performing indicator for pathogenicity and benignity has not changed from the original benchmark.

All files used for this benchmark were uploaded for review as:

- **TAPES_validation_synthetic.vcf**: the synthetic vcf created from ClinGen data

- **TAPES_validation.charger.txt:** CharGer’s output

- **TAPES_Validation.intervar.txt**: InterVar’s output

- **TAPES_Validation.tapes.txt**: TAPES output

- **Supplementary_Table_3_TAPES_Validation.full.txt:** All software predictions and expert panel decision in one file **\# Textual comments

Line 35: "does not take into account the abundance of a variants in a cohort" should be "do not take into account the abundance of variants in a cohort"**

Author’s answer: The sentence has been modified to read “*do not take into account the abundance of variants in a cohort*”. **Line 66-70. I could not perform the described steps without installing ANNOVAR and the setting up the database. Either include clearly in the manual how this can be done or modify this paragraph.**

Author’s answer: This sentence (“*TAPES sorting option can be used with both ANNOVAR and VEP annotated variant calling files (VCF). However we also provide users with simple wrapping tools for a local installation of ANNOVAR to simplify the workflow. Users can annotate VCF, gzipped VCF and binary VCF (BCF) using two simple commands without having to specify the databases and annotations to use.*”) clearly indicates that TAPES provides ANNOVAR wrapping for vcf annotation. This obviously requires users to install ANNOVAR but is completely distinct from TAPES main sorting and prioritising function (which can function without ANNOVAR). A sentence was added line 68 to better reflect that fact. **Line 116 should read "TAPES provides an array of different useful reports."**

Author’s answer: The sentence is now corrected. **Line 119. "on the command line" not "in"**

Author’s answer: The sentence is now corrected. **Line 121. "a pathway" not plurar**

Author’s answer: The sentence is now corrected. **Line 122. "users do research" could be "run searches"**

Author’s answer: The sentence is now corrected. **Line 145. TAPES will or does?**

Author’s answer: The sentence is now corrected to ‘*TAPES provides an appropriate warning for all of those cases*’. **Line 158. Is the table only used as reference or also as input for the analysis? Please add the input.**

Author’s answer: The table was used as an input for the benchmark. The sentence has been modified to reflect that fact. This table has been uploaded as **CharGer_and_Panel_Benchmark.xlsx .
Line 167-168. How do the ROC curves suggest the threshold values?**

Author’s answer: The ROC curves are plotted by using all possible thresholds across a range and plotting the True positive rate and false positive rate.

| Benignity | | |  | Pathogenicity | | |
| --- | --- | --- | --- | --- | --- | --- |
| Threshold  TAPES probability | True positive Rate | False positive rate |  | Threshold  TAPES probability | True positive Rate | False positive rate |
| 0 | 0.003164557 | 0 |  | 0 | 1 | 1 |
| 0.05 | 0.268987342 | 0 |  | 0.05 | 1 | 0.731012658 |
| 0.1 | 0.528481013 | 0.015748031 |  | 0.1 | 1 | 0.674050633 |
| 0.15 | 0.528481013 | 0.015748031 |  | 0.15 | 0.984251969 | 0.471518987 |
| 0.2 | 0.689873418 | 0.023622047 |  | 0.2 | 0.976377953 | 0.310126582 |
| 0.25 | 0.689873418 | 0.023622047 |  | 0.25 | 0.976377953 | 0.310126582 |
| 0.3 | 0.689873418 | 0.023622047 |  | 0.3 | 0.976377953 | 0.310126582 |
| 0.35 | 0.797468354 | 0.070866142 |  | 0.35 | 0.929133858 | 0.202531646 |
| 0.4 | 0.797468354 | 0.070866142 |  | 0.4 | 0.929133858 | 0.202531646 |
| 0.45 | 0.797468354 | 0.070866142 |  | 0.45 | 0.929133858 | 0.202531646 |
| 0.5 | 0.882911392 | 0.118110236 |  | 0.5 | 0.881889764 | 0.117088608 |
| 0.55 | 0.882911392 | 0.118110236 |  | 0.55 | 0.881889764 | 0.117088608 |
| 0.6 | 0.882911392 | 0.118110236 |  | 0.6 | 0.881889764 | 0.117088608 |
| 0.65 | 0.882911392 | 0.118110236 |  | 0.65 | 0.881889764 | 0.117088608 |
| 0.7 | 0.974683544 | 0.196850394 |  | 0.7 | 0.803149606 | 0.025316456 |
| 0.75 | 0.974683544 | 0.196850394 |  | 0.75 | 0.803149606 | 0.025316456 |
| 0.8 | 0.974683544 | 0.196850394 |  | 0.8 | 0.803149606 | 0.025316456 |
| 0.85 | 0.993670886 | 0.251968504 |  | 0.85 | 0.748031496 | 0.006329114 |
| 0.9 | 0.996835443 | 0.307086614 |  | 0.9 | 0.692913386 | 0.003164557 |
| 0.95 | 0.996835443 | 0.307086614 |  | 0.95 | 0.692913386 | 0.003164557 |

ROC curves used FPR and TPR calculated for every threshold and plots the result. We picked those thresholds because they ensured a relatively high TPR while keeping the FPR relatively low.

Based on the validation dataset, we now provide two sets of recommended thresholds (lenient (yellow) and strict (green)).  **Figure 3: Why is the old TAPES curve used instead of the new one that is already in the git repo?**

Author’s answer: the ‘new’ method on the github repository, has been developed after the original submission of the manuscript. We feel that the original method must first be published before we can refine it properly because the ‘new method’ is based on the original one (with adjustments).

We believe that the original method for calculating OR from MAF is strong and valid albeit too stringent.  **\# Code review
The code still contains bugs that cause it to crash. Although the git repo suggests using `python tapes.py`, since tapes.py is written in python3 and the default python on many linux systems is python2 the program crashes.**

Author’s answer: The repository/wiki has been changed to only include “python3” in the commands since “python2” is indeed still the default for many linux systems and “python3” is universal. **Many of the example commands contains incorrect hyphen character that results in an error when copy pasting them to command line.**

Author’s answer: All hyphens has been corrected. It was due to auto-correcting from hyphens (-) to en dash (–)

**Attempting o run the "Quick Start" section

`python tapes.py db -s -A /path/to/annovar/` -> `python3 tapes.py db -s -A ~/temp/tapes/annovar/`

Worked fine with absolute path, but fails with relative path with a non informative error.

`python3 tapes.py db -s -A ../tapes/annovar/` Gives the following output:
```
No acmg_db path given and no db_config.json found
Default is: /home/user/temp/tapes-0.1/acmg_db

***TAPES: SEE DATABASE***

2019-09-04 13:45:59.....Fetching ANNOVAR Alldb file
NOTICE: Web-based checking to see whether ANNOVAR new version is available ... Done
NOTICE: Downloading annotation database http://www.openbioinformatics.org/annovar/download/hg19_avdblist.txt.gz ... OK
NOTICE: Uncompressing downloaded files
NOTICE: Finished downloading annotation files for hg19 build version, with files saved at the '.' directory
NOTICE: Web-based checking to see whether ANNOVAR new version is available ... Done
NOTICE: Downloading annotation database http://www.openbioinformatics.org/annovar/download/hg38_avdblist.txt.gz ... OK
NOTICE: Uncompressing downloaded files
NOTICE: Finished downloading annotation files for hg38 build version, with files saved at the '.' directory
Traceback (most recent call last):
File "tapes.py", line 406, in
tf.check_online_annovar_dbs(annovar_path)
File "/home/user/temp/tapes-0.1/src/t_func.py", line 883, in check_online_annovar_dbs
with open(outfile_hg19, 'r') as file:
FileNotFoundError: [Errno 2] No such file or directory: '../tapes/annovar/hg19_avdblist.txt'
```**
Author’s answer: This bug was corrected by transforming all paths to absolute paths at the beginning of the script (saving the absolute path of ANNOVAR in the config file “tapes/src/db_config.json”).

**`python tapes.py db -b --acmg --assembly hg19` -> `python3 tapes.py db -b --acmg --assembly hg19` Fails
```
No acmg_db path given and no db_config.json found
Default is: /home/user/temp/tapes-0.1/acmg_db

***TAPES: DOWNLOAD DATABASE***

No annovar path given and no db_config.json found
Traceback (most recent call last):
File "tapes.py", line 358, in
tf.build_annovar_db(annovar_path, args.assembly, args.acmg)
NameError: name 'annovar_path' is not defined
```**

**`python3 tapes.py annotate -i toy_dataset/toy.vcf -o test/output.vcf --acmg –a hg19` does not run and prints out the help page plus the following warning:
```
tapes: error: unrecognized arguments: –a hg19
```
After changing the hyphen to the correct character ` python3 tapes.py annotate -i toy_dataset/toy.vcf -o test/output.vcf --acmg -a hg19`

```
No acmg_db path given and no db_config.json found
Default is: /home/user/temp/tapes-0.1/acmg_db

***TAPES: ANNOTATE***

No annovar path given and no db_config.json found
Traceback (most recent call last):
File "tapes.py", line 384, in
tf.process_annotate_vcf(args.input, args.output, annovar_path, args.assembly, args.ref_anno, args.acmg)
NameError: name 'annovar_path' is not defined
```**
Author’s answer: All “annovar_path is not defined” errors are now fixed by saving the absolute path of ANNOVAR in the config file (tapes/src/db_config.json) after the first setup. This error can also be bypassed by using the “-A /path/to/annovar/” in the command.

**`python3 tapes.py sort -i toy_dataset/toy_annovar_multi.vcf -o test-sort/ --tab` works and creates a folder with three plots (png) and `test-sort.txt`, which is a tab separated file

`python3 tapes.py analyse -i test-sort/test-sort.txt -o test-report/report.txt --single_option` Fails with the following error:

```
tapes: error: unrecognized arguments: --single_option
`python3 tapes.py analyse -i test-sort/test-sort.txt -o test-report/report.txt` Runs without error, but creates no output
```
No acmg_db path given and no db_config.json found
Default is: /home/user/temp/tapes-0.1/acmg_db

***TAPES: RE-ANALYSE***

2019-09-04 14:07:10.....48 samples found
2019-09-04 14:07:10.....Output type: TXT/TSV + XLSX
2019-09-04 14:07:10.....Done
```

However, `python3 tapes.py sort -i toy_dataset/toy_annovar_multi.vcf -o test-full/ --tab --by_gene --by_sample --enrichr --list "MLH1 MSH6 MSH2" --disease "autosomal dominant" –kegg "pathways in cancer"` does work.**

Author’s answer: This error was caused by a bad explanation for the function analyse. “--single_option” was a placeholder command used to reflect the fact that “analyse” will only accept a single option for analysis. Using no option will run the command without creating any output.

Analyse is a convenience option to get side-reports (by-gene, by-sample, filters, etc) without having to produce the main report again.

Available options for re-analysis of the main output are “--by_sample”, “--by_gene”, “--list”, “--kegg” or “--enrichr”.

The wiki have been updated for clarity.

**Additional comments:** The PDF version of the instruction manual was dropped. All documentation can now be found on the github wiki, which was reworked thoroughly. However, an offline version of the wiki compiled into a PDF file automatically is provided with the repository. This allows for more flexibility and easier automation.

Additionally, a new release (0.1.1) has been added to correct all bugs and code reworks done after the reviewer’s comments.

I appreciate all the helpful comments from the reviewers and trust the manuscript is ready for publication in PLOS Computational Biology.

Kind regards,

Alexandre Xavier

PhD Candidate (Medical Genetics)

University Of Newcastle

School of Biomedical Science and Pharmacy
